# Comparative Assessment of the Adhesion Forces of Soft Silicone Materials to the Denture Base Material (PMMA) Conditioned with Sandblasting

**DOI:** 10.3390/ma17143439

**Published:** 2024-07-11

**Authors:** Amadeusz Kuźniarski, Weronika Huss, Tomasz Dąbrowa, Edward Kijak

**Affiliations:** 1 Department of Prosthetic Dentistry, Wroclaw Medical University, 50-425 Wrocław, Poland; amadeusz.kuzniarski@umw.edu.pl (A.K.); tomasz.dabrowa@umw.edu.pl (T.D.); 2 Faculty of Mechanical Engineering, Wroclaw University of Science and Technology, 50-370 Wrocław, Poland

**Keywords:** adhesion acrylic dentures, surface development, soft relining materials

## Abstract

Background: In patients undergoing surgery for oral cancer, soft support materials are used to minimise trauma to the soft tissues. Silicone-based liners are widely used in prosthetic dentistry. A prerequisite for long-term Adhesion of the liner to the denture base is largely dependent on the surface preparation of the denture material. Objectives: The aim of the present study was to investigate whether surface preparation of the acrylic material by sandblasting increases the adhesion of the silicone support material to the acrylic denture plate. Material and Methods: The study included adhesion testing of four silicone-based soft cushioning materials (Silagum Comfort, Elite Soft Re-lining, Ufi Gel SC, Mucopren Soft) on a total of 270 samples. Each material was tested on 15 samples. Three subgroups with different surfaces were separated: 1 raw—standard surface treatment with a cutter, and 2 sandblasted, with 100 and 350 µm alumina grain at 90°. The samples were subjected to seasoning: 24 h and six weeks. The adhesion force of silicone to acrylic was measured by performing a tensile test using a universal two-column testing machine. Results: The highest bond strength was recorded for Silagum on the surface prepared using 100 µm abrasive and seasoned for 6 weeks (291.5 N). The smallest among the maximum forces was recorded for the Mucopren material (81.1 N). For the Mucopren system with a raw and sand-blasted surface (350 µm), the adhesion strength increased after six weeks. In contrast, the durability of the joint decreased for the 100 µm sandblasted surface. The Elite material exhibited similar values for maximum forces (271.8 N) and minimum forces (21.1 N). The highest strength (226.1 N) was recorded for the sample from the group prepared with 350 µm abrasive and seasoned for 24 h. The lowest value (72.6 N) occurred for the sample from the group with 100 µm abrasive and seasoned for 6 weeks. Conclusions: Sandblasting of acrylic plastic improves adhesion to selected relining silicones. 2. The size of the abrasive employed has an impact on the adhesion between the acrylic plastic and the bedding silicone. 3. In the case of some relining systems (Mucopren), an increase in roughness through sandblasting has the effect of reducing the durability of the bonded joint.

## 1. Introduction

**Figure 1:**
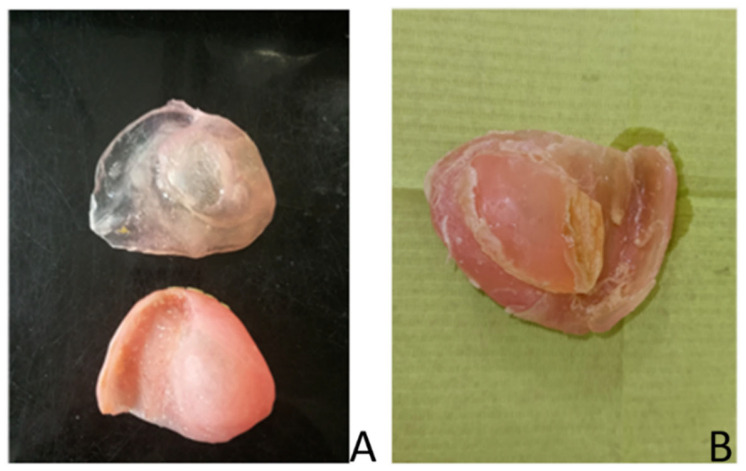
(**A**) Separation of the support material into a whole; (**B**) loss of marginal tightness—change in the properties of the support material (after two months of use).

Oral and oropharyngeal cancers represent a significant epidemiological problem worldwide. According to the World Health Organization, they rank seventh among all registered cancers [1]. They account for approximately 5% of all those diagnosed [2]. In Poland, cases of malignant tumours of the oral cavity represent 4% of males and 1% of women of all cancers, according to the National Cancer Registry [3]. Every year, several thousand new cases of this disease are detected. A significant increase in the incidence of oral mucosa lesions and oral cavity tumours in a younger age group of patients has been reported. Unfortunately, the increase in these cases, for a variety of reasons, continues to trend upwards. Oral HPV infections are considered to be the reason for the increase in cases among increasingly younger individuals [4]. The treatment of choice for these patients is to undergo more or less extensive surgery with subsequent prosthetic treatment. In non-operated patients with a healthy mouth, rehabilitation with complete dentures is considered satisfactory by most patients. However, it can also present several functional problems, including persistent pain, inability to chew and discomfort, and a general deterioration of quality of life [5]. The use of post-operative acrylic removable complete and partial dentures can cause significant difficulties for patients. PMMA is the primary material used in dental prosthetics, but not only. Generally in medicine, it is used to make dressing materials, contact lenses, endoprostheses in plastic surgery, synthetic tissue adhesives, or intraocular lenses. Pain and discomfort often persist for months. The objective of the patient’s prosthetic supply is to regain the physiological function of the vital organ damaged by the surgery. The traumatising effect of the prosthesis plate on the mucosal bone substrate can be reduced by evenly distributing the loading forces [6]. The oral cavity can be isolated from adjacent spaces (nasal cavity) by using obturators. Soft relining materials are used to achieve the least amount of soft tissue traumatisation. The pressure-sensitive soft relining layer is designed to facilitate prosthetic treatment by enhancing the comfort of the prostheses in use, reducing the adaptation period to the prosthesis [7,8,9,10]. These methods have demonstrated the potential to enhance muscular activity, improving chewing performance with soft liners. Another significant benefit of these materials is their ability to create a tight seal between the oral cavity and other surrounding structures, such as for example the nasal cavity. Currently, the most used soft materials for long-term relining can be divided into two main groups based on their chemical composition: silicone and acrylic [11,12]. The results of numerous authors demonstrate that acrylic materials exhibit significantly less stability in terms of hardness over time in comparison to silicone materials [13,14,15,16,17]. The primary issue with the utilisation of this group of acrylic materials is their hardening, which is a consequence of the direct leaching of the plasticizer in the oral environment. The material, exhibiting inferior elasticity, is unable to retain its therapeutic properties [18]. In contrast, silicone materials do not contain plasticizers, and their elastic properties are attributed to their internal structure, thus ensuring an overall stable hardness throughout the utilisation period The fundamental component of silicones is polydimethylsiloxane with hydroxyl groups. Silicones can undergo polymerisation under heat (HTV) or at room temperature (RTV). A group of hot-polymerising silicones exhibits superior mechanical properties and enhanced adhesion to acrylic materials [19]. Nevertheless, despite the enhanced long-term elasticity retention, silicones utilised for lining are not optimal materials. Furthermore, these materials lose their properties over time. Since they do not chemically bond to the acrylic denture plate, delamination and separation of the silicone material from the denture plate in part or in whole becomes a problem (Figure 1A,B).

The discovery of a gap between the denture plate and the relining material is indicative of the necessity to remove the soft support material and to repeat the framework procedure [20]. This prevents the accumulation of bacterial plaque and enables improved comfort and function for the patient, particularly those who use a denture with an obturator.

It is evident that the loss of the properties of the lining is contingent upon a multitude of factors, including the patient’s diet (pH), the composition of saliva, the presence of inflammation in the oral cavity, and the means and methods of hygiene employed. In addition to compromising the properties of the lining materials, the adhesion of the liner to the denture plate is largely influenced by the preparation of the surface of the denture material. In accordance with industry standards, manufacturers of soft materials for relining recommend working the surface of the acrylic with a dedicated cutter and applying a chemical bonding agent (primer or adhesive) to the surface. Nevertheless, the manufacturer of Ufi Gel material does not recommend sandblasting as a method of increasing adhesion surface area. However, the instructions do not specify the parameters to be employed in this process.

Accordingly, the authors of the present study set out to achieve the following objectives: the objective of this study was to investigate whether the preparation of the surface of the acrylic material by sandblasting increases the adhesion of the cushioning silicone material to the acrylic denture plate. Furthermore, if this is the case, we also sought to ascertain whether there are universal parameters that could be recommended for practical clinical management.

## 2. Materials and Methods

### 2.1. Materials Subjected to the Tests

For the study, 4 materials used for long-term soft linings of removable complete and partial dentures with obturators were selected (Table 1). All tested materials were silicone based: Silagum Comfort (DMG, Hamburg, Germany), Elite Soft Relining (Zhermack S.p.A., Badia Polesine, Italy), Ufi Gel SC (VOCO, Cuxhaven, Germany), and Mucopren Soft (Kettenbach GmbH, Eschenburg, Germany). Tests on the adhesion strength of the cladding materials to the acrylic material were performed in accordance with the European standard ISO 10139-2 [21].

### 2.2. Preparation of Samples for Testing

**Figure 2:**
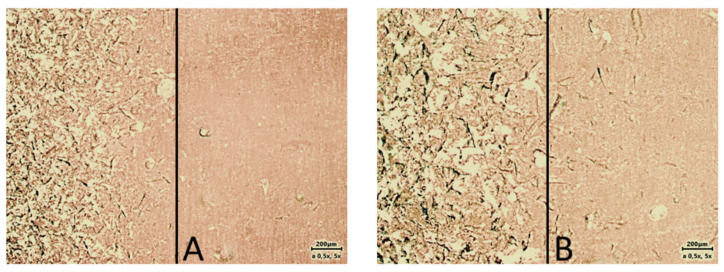
Image of surface prepared for testing (**A**) sandblasted with 100 µm grit (left side) vs. raw surface (right side); (**B**) sandblasted with 350 grit vs. raw surface (right side).

PMMA (polymethylmethacrylate) acrylic resin, Vertex Rapid Simplified (Vertex Dental BV, Soesterberg, The Netherlands), was used as the base material for the study. This material is hot-polymerized and is used to produce plates for complete and partial dentures. Plates measuring 2 cm × 2 cm and 2 mm thick were made from this material, according to specifications and requirements. Cylindrical retainers with a length of 3 cm and a diameter of 1 cm were attached to the manufactured plates with adhesive bonding. The active surfaces of the acrylic plates were machined with cutters (according to the recommendations of the substrate material manufacturers). Then, the plates, made of PMMA and fitted with a holder, were sandblasted with 100 and 350 µm abrasive at 90 and a 2-bar jet. The quality of the three different sur-faces used in the study was evaluated using additional acrylic test plates that had been half-sandblasted. Figure 2 shows the differences between the raw and sandblasted surfaces. These were observed under a Zeiss Axio Lab Mat microscope with a microscope camera transmitting images to a computer from Carl Zeiss Microscopes GmbH (Jena, Germany). Each of the test materials was applied to the surface of the properly prepared plates (in the central part of the plates), with the material placed in a special ring with a diameter of 10 mm and a height of 3 mm. Polymerization of samples of all materials was carried out according to the manufacturers’ recommendations. After polymerization of the silicone, the auxiliary ring, which prevented excessive spreading of the silicone under test, was again removed, thus obtaining a sample ready for testing—silicone material with a diameter of 10 mm and a height of 3 mm between two acrylic plates.

### 2.3. Create Groups of Test Materials 

The test material was divided into 3 basic groups: 1 raw and 2 sandblasted for each test material. All samples were seasoned in distilled water in a greenhouse at a constant temperature of 37 ± 1 °C. This was objective to avoid the influence of possible chemical compounds and free ions found in other solutions. The temperature corresponded to that found in the oral cavity. Each set prepared for testing contained 15 samples in the following configurations: raw, sandblasted 100 µm, and sandblasted 350 µm—seasoned 24 h and 6 wks.

### 2.4. Strength Tests of Lining Materials

**Figure 3:**
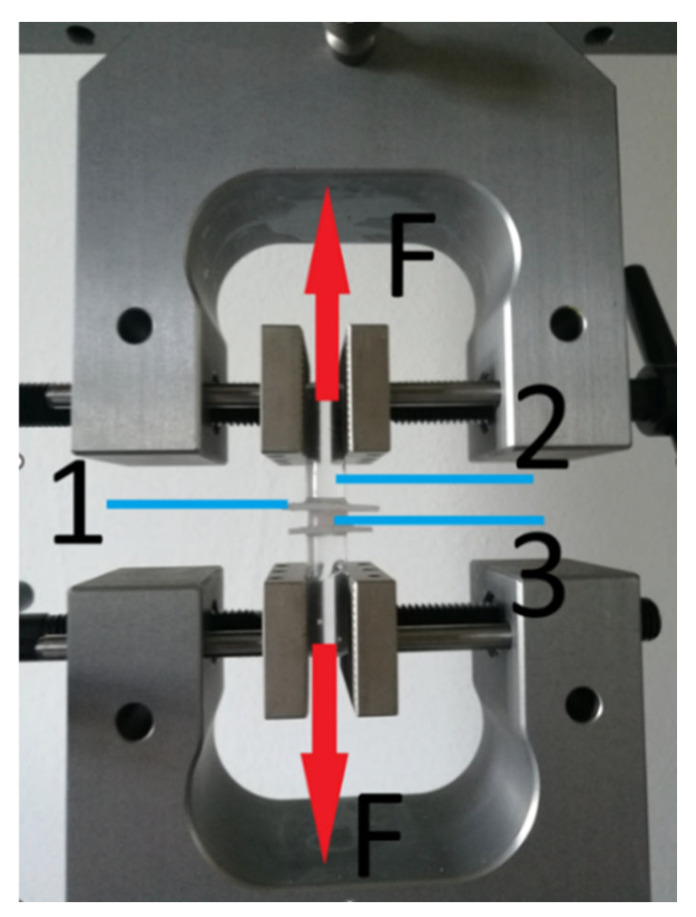
Schematic of the construction and mounting of the specimen in the testing machine: 1—acrylic plate; 2—mounting bracket; 3—cylindrical silicone; F—force and direction of action.

A total of 360 specimens were thus prepared for testing, which were subjected to tensile tests at 10 mm/min on a Z 10-X700 dual-column testing machine from Thumler GmbH (Nurnberg, Germay). The test scheme is shown in Figure 3. The most popular and reliable method, thanks to the standardisation of the European Standard ISO 10139-2 [21], is the tensile strength test. The test consists of stretching the sample axially at a constant speed at room temperature until it breaks or ruptures completely.

**Figure 4:**
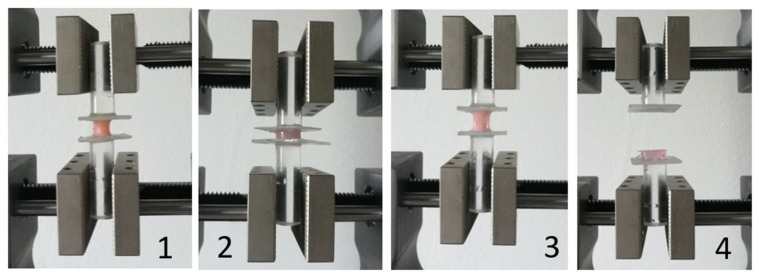
Tested materials (different phases) during tensile test: 1—Silagum Comfort; 2—Elite Soft Relining; 3—Ufi Gel S.C.; 4—Mucopren Soft—adhesive separation from PMMA plate.

Figure 4 presents the course of the experiment of individual materials in various phases until the complete adhesive separation of the tested silicone from the acrylic plate.

### 2.5. Statistic Tools

All calculations were conducted using Matlab^®^ 2023 from The MathWorks, Ltd. (Cambridge, UK).

In all of presented statistic tests, a standard significance level of α = 0.05 was used.

#### 2.5.1. The Kruskal–Wallis Test

The most general test that can be performed without assuming the normality of distributions is the non-parametric Kruskal–Wallis test. This test is used for more than two samples. The null hypothesis of this test is that all samples come from populations with the same distributions. However, if this hypothesis is rejected, it does not indicate which of the samples shows a significant difference. This Kruskal–Wallis test was performed on samples within each limiting material group. 

#### 2.5.2. The One-Way ANOVA Test

One of the most widely used statistical tools to analyse phenomena depend on one factor in more than two samples in one-way ANOVA. It is a kind of a parametric test. The comparison is made by comparing mean values in all studied samples.

The ANOVA null hypothesis is as follows:

(1) $$H_{0}:\mu_{1}=\mu_{2.}=\cdot \cdot \cdot =\mu_{k}$$

where:

*μ*_1_—mean durability of glue joint for a sample no 1;
*μ*_2_—mean durability of glue joint for a sample no 2;
*μ_k_*—mean durability of glue joint for a sample no k;
*K*—any number of studied samples.

There is just one (two-sided) alternative hypothesis:

(2) $$H_{0}:\mu_{1}\neq \mu_{2}\neq \cdot \cdot \cdot \neq \mu_{k}$$

As can be seen from the above, the rejection of the null hypothesis in favour of the alternative results only in a conclusion that not all mean values are equal. There is no information on which values are different. More precise analysis is made by using other statistical tests.

There are some assumptions that populations should meet to allow for ANOVA testing, as follows:

- Samples are independent.
- Homogeneity of variance in all populations from which the samples are derived—the Brown-Forsythe test is used to verify this assumption.
- Normality of residuals (deviations from the mean)—for this purpose, the Jarque–Bera test is most often used.

The above tests were conducted for all samples from Table 3 and none of them rejected the null hypothesis of homogeneity of variance or normal distribution. Therefore, it is possible to use the ANOVA test to verify the given problems.

#### 2.5.3. The One-Tailed *t*-Test

A similar test to ANOVA, but for a case of two samples (of small size), is the two-tailed *t*-test for two mean values. The assumptions for using this test are the same as for ANOVA and, as already mentioned, they are met.

However, more information on the relations between mean values are given by one-tailed variants of this test. That is why they were chosen to carry out the analyses. The structure of one-tailed *t*-tests are as follows:

The null hypothesis:

(3) $$H_{0}:\mu_{1}=\mu_{2}$$

The alternative hypotheses:

(4) $$\mathrm{right-tailed}:\;\;\;H_{1}:\mu_{1}>\mu_{2}$$

(5) $$\mathrm{left-tailed}:\;\;\;H_{1}:\mu_{1}<\mu_{2}$$

A possible rejection of the null hypothesis in favour of one-tailed hypothesis provides additional information about which of the mean value is greater.

#### 2.5.4. Determination of the Size of Differences between Samples (Effect Size)

When a null hypothesis is rejected, it may be interesting to consider how large the difference is between tested quantities which led to this situation. It may be estimated using the so-called effect size. It is calculated according to a statistical test that was used. In case of the ANOVA and *t*-test, a Cohen’s d is used [22]. It is calculated by a formula:

(6) $$d=\sqrt{\frac{\left(n_{1}-1\right)s_{1}^{2}+\left(n_{2}-1\right)s_{2}^{2}+\ldots +\left(n_{k}-1\right)s_{k}^{2}}{n_{1}+n_{2}+\ldots +n_{k}-k}}$$

where:

*n*_1_, *n*_2_, …, *n_k_*—size of all tested samples.
$s_{1}^{2},\;s_{2}^{2},\ldots ,\;s_{k}^{2}$—variances of all tested samples.

Afterwards, the Cohen’s d can be compared to values suggested in [23], and is presented in Table 2. This gives an orientation into how large the differences are, especially when more than two samples are available.

## 3. Obtained Results and Analysis

### 3.1. Results

Statistical analysis revealed clear correlations between the strength of the adhesive bond between the relining silicone and the acrylic plate with a different surface finish. In light of the above, the following lines of analysis were formulated:

1. Does the strength of the bonded connection depend on the seasoning time of the specimen? In this experiment, specimens were seasoned for either 24 h or six weeks.
2. Does the strength of the bonded joint depend on the acrylic surface finish?

In this issue, samples with three degrees of surface finish were used: raw surface, and surfaces sandblasted with abrasive of 100 µm and 350 µm gradation. We analysed four different commercially available silicone + adhesive systems for these issues.

The highest bond strength was recorded for Silagum on the surface prepared using 100 µm abrasive and seasoned for 6 weeks (291.5 N). Conversely, the lowest value for this material (24.4 N) was recorded for samples with the same abrasive, but seasoned for 24 h.

The Elite material has similar maximum and minimum force values (271.8 N and 21.1 N, respectively). The maximum value was recorded for a control sample seasoned for six weeks, and the lowest for the sample with a surface treated with 100 µm abrasive and seasoned for 24 h.

The Mucopren material recorded the smallest maximum force (81.1 N). This value applies to a sample from the control group, seasoned for 24 h. This group also has the highest average force value (63.2 N) in all Mucopren groups. This indicates that for the Mucopren material, increasing the surface roughness is detrimental to the durability of the connection. This is further evidenced by the fact that one of the samples from the group prepared with 350 µm abrasive and with a 6-week seasoning time has the lowest connection strength (29.4 N), with the lowest average also in this group of samples (45.9 N).

The highest strength was recorded for the Ufi Gel material with a sample from the group prepared with 350 µm abrasive and seasoned for 24 h (226.1 N). The lowest value (72.6 N) was recorded for the sample from the group with 100 µm abrasive and seasoned for 6 weeks.

During the tensile strength test, the bonded joint did not fail. Instead, there was a rupture of the silicone component. Such specimens were immediately rejected from the analysis on the durability of the bonded joint. This is because in this type of testing, the “destruction” of the material can be adhesive, cohesive, or mixed. This study only tested for adhesive rupture, so the results of the analysis of samples meeting the condition are as follows in Table 3.

### 3.2. Thesis I. The Seasoning Time of the Glueing Joint Causes a Change in the Adhesive Strength

First of all, the Kruskal–Wallis test was performed on samples within each lining material. The *p*-values obtained in this way are presented in Table 4. For each material, these values are below the standard significance level (α = 0.05). Therefore, already, at this stage, it can be concluded that the variety of abrasive granulation or the seasoning time affected the strength of the connection.

In order to verify thesis I, samples with the same surface finish but with different seasoning times had to be compared with each other. There is only two cases within one surface finish group for every system (24 h and 6 weeks). Therefore, one-tailed *t*-tests were employed to compare two mean values.

*p*-values derived for every pair of samples are presented in Table 5. *p*-values which led to a rejection of the null hypothesis are marked with *. These rejections were made due to the according alternative hypothesis. 

The tests show that the timelapse does not have the same effect on the strength of adhesives of all brands. In case of Elite system, the null hypothesis was not rejected in most test variants. This may indicate the lack of time influence in joint durability, at least in the examined time horizon (6 weeks). 

For any surface preparation in the Mucopren system, the null hypothesis was rejected in favour of the left-tailed hypothesis. This means that, most probably, the durability of the glued joint rose in 6 weeks’ time.

Results for Silagum and Ufi Gel systems did not allow for clear interpretation.

The next step was to closer examine the rejected cases to estimate the effect size, which caused this rejection. The results are in Table 6. The interpretation was made according to values presented in Table 3. It is worth noticing that the seasoning period does not influence the durability of the glue in same way. 

For the Mucopren system with a raw and sandblasted surface (350 µm), the adhesive strength increased after 6 weeks, although not to a similar extent, whereas in the case of the surface sandblasted with 100 µm, the durability of joint decreased. 

As for the Silagum system, for the surface treated with the abrasive of 100 µm, the hypothesis about the lack of changes in durability during the seasoning period could not be rejected, whereas for the surface of 350 µm, the force increased. For the Ufi Gel system, this relation is opposite. 

### 3.3. Thesis II. The Surface Finish Increases the Glued Joint Durability

To verify this assumption, samples were separated into groups of different surface finish, but same seasoning time. This way allows for examination of the influence of the surface without taking into account the seasoning time.

There are more than two samples within most groups, so ANOVA was used to perform this analysis. The Silagum and Ufi Gel groups consist of two samples (100 μm and 350 μm), so the two-tail *t*-test for two mean values were used. The *p*-values obtained in these tests are presented in Table 7.

This preliminary analysis shows that only for the Elite system the null hypothesis of durability equality for every kind of surface and time lapses was not rejected. In order to analyse the remaining cases more precisely, it was necessary to carry out one-tailed *t*-tests between individual pairs of samples (Table 8) and to estimate their effect size (Table 9).

According to assumed intervals (Table 3), all variants of sample comparison due to surface finish have large or very large effect size. This proves the significant influence of the surface finish quality on the strength of the glued joint. In some cases shown in Table 9 the effect size is negative. This indicates the opposite effect of increasing the roughness of the bonded surface. The conclusion is that for some systems, increasing the roughness of sanding reduces the durability of the glued joint.

## 4. Discussion

Inadequate bonding of the lining materials poses a problem for both the dentist and the patient using the prosthesis, which requires lining with soft material. Obturating prostheses used in patients who have undergone surgical procedures for non-neoplastic conditions must meet exceptional requirements. They are used in special conditions, so they must be exceptional. Such patients are more susceptible to chemical and physical factors, including increased salivation or xerostomia, various disinfectants used on a larger scale, local inflammatory, fungal infections, drugs, and so on [24,25]. The poor adhesion of silicones used for this purpose is since it is largely a chemical-mechanical combination. For many years, research has been underway to find an effective antidote to solve this problem. However, many researchers believe that modifying the prosthesis plate by developing its surface using various methods will sufficiently increase the mechanical retention of both materials.

When using silicone-based soft inserts, it is crucial to use a suitable adhesive. Without it, there will be no chemical bond between the two materials established [26,27]. This is undoubtedly the case. Without proper conditioning of the PMMA plate surface, the silicone will almost immediately separate from the prosthesis plate, as shown in Figure 1A. In our study, we strictly followed the manufacturer’s instructions for preparing the PMMA plates. We observed the method and time of application. We therefore disagree with Mese’s assertion that when chemical bonding does not occur, this is the main cause of adhesive-type damage [28]. We focused on this type of damage, but it is important to note that cohesive and adhesive-type damage also occurred in each group, despite the same conditions and parameters being applied to all the samples. In earlier inquiries, Bayati et al. found that micropores and air bubbles formed at the interface between the surface of the denture base material and the soft silicone-based liner, which were not visible to the naked eye [29]. This was confirmed by their study conducted with a scanning electron microscope (SEM). The authors of this study have concluded that there is a chemical reaction between the solvent contained in the primer (ethyl acetate) and the base material of the prosthesis. This causes the formation of air inclusions, which act as a site of crack initiation and reduce the contact area and bonding strength. This conjecture is largely supported by our findings. In general, the smallest retention force of silicone on the base material was obtained for the surface sandblasted with a large grain (350 µm). This was observed for only one silicone material (Ufi Gel), but after six weeks of seasoning, this force was higher. It is therefore likely that the larger the diameter of the abrasive, the larger the voids are closed between the liner and the base material, and that the total size of these voids reduces the surface area of adhesion and thus the strength of the bond. 

In our study, we applied all materials to the baseplate using a mixing tip directly from the factory cartridge, without additional mechanical steps. It is therefore clear that not only the size of the cavities in the surface after sandblasting affects the area of adhesion between the two materials, but also the initial viscosity of the silicone used for linings. The higher the likelihood of “closing” the voids between the materials. Kim et al. [13] were the first to highlight this problem. In their study, GC Reline Ultrasoft and Mucopren Soft had significantly lower average tensile bond strengths, respectively. The results showed that GC Reline Ultrasoft had the lowest average tensile bond strength at 0.82 ± 0.32 MPa, while GC Reline Soft had the highest at 2.99 ± 0.43 MPa.

In contrast, in the study by Wyszyńska et al., the GC Reline Soft material also stood out among all the silicone materials tested. After one week of seasoning, they showed a high average bond strength of 2.15 MPa. The authors also reported that the decrease in average bond strength over time was not significant, ranging from 2.9% (Mollosil Plus) to 14.6% (Elite Soft Relining), which, for the latter material, coincides with our observations [30].

They found that GC Reline II Soft is mixed with mixing tips to avoid visible traces of air inclusion inside the mixed material. With careful application to the surface, air can be avoided at the interface between the two materials. However, they do not mention the possibility of air being enclosed, for example, in the cavities created after sandblasting. It is also important to note that chemical surface preparation of PMMA (in addition to the manufacturer’s indications) can significantly enhance the adhesion strength of the silicone liner. Special chemical bonding agents, which are a mixture of appropriately selected solvents and reactive polymers, are responsible for increasing wettability, which significantly improves the adhesion of the lining material to PMMA [31,32]. Cavalcanti et al. demonstrated in their study that additional surface treatment with methyl methacrylate and ethyl acetate significantly improves the adhesion of the flexible silicone-based prosthesis liner to PMMA [33]. However, they did not find a correlation between surface properties and tensile bond strength. We disagree. Our study proves that such a correlation exists From the data available in the literature, different test methods have been used in similar tests, including shear, bond strength, peel bond, and tensile bond strength tests. However, the tensile bond strength test is the most used, as demonstrated by Wemken et al. furthermore, the authors of this study have found that bond strength is not affected by either the base material or the pretreatment [34]. Other studies have used different test methods, making it difficult or impossible to compare different tests. Więckiewicz et al. found that, regardless of the testing techniques used, commercially available silicones used for lining give satisfactory results in terms of tensile and shear strength. They can therefore be recommended for clinical use, which our observations also confirmed. Their research revealed that silicone-based relining materials exhibited varying shear bond strength values. Elite Super Soft demonstrated an average of 0.67 MPa, while A-Soft Line 30 exhibited an average of 1.32 MPa. The values of the forces are similar, but they cannot be directly related to ours because they were shear forces [35]. The authors are correct in stating that only certain conclusions can be determined with a single investigation.

It is not possible to make a universal and indisputable determination of the bond strength between additive and subtractive denture base materials on the one hand and soft denture inserts on the other in the current state of research and knowledge. It is possible to improve the bonding strength and breaking strength of soft lining materials by flowing the material into the microporosity after sandblasting. However, air voids may form, reducing the adhesion strength and durability of this bond. The lack of development of universal procedures for handling the cases in question is clear evidence that further research is needed in this area.

## 5. Limitations of the Study

Our study, like most of those cited here, has some limitations, as follows: the samples were stored in distilled water and not under simulated oral conditions, no physical forces were applied to the resting samples (simulated use), and we did not examine cross-sections of the joints for air bubbles or unfilled microspaces. We also did not test the elasticity (hardness) between the different materials tested.

## 6. Conclusions

The results obtained during the study allow us to draw the following conclusions:

1. Sandblasting of acrylic plastic improves adhesion to selected relining silicones.
2. The size of the abrasive used affects the adhesion of acrylic and silicone. The use of too large an abrasive can weaken the strength of adhesion.
3. For some relining systems (Mucopren), sandblasting reduces the bond strength of the silicone to PMMA.

## Figures and Tables

**Table 1:** Materials used for the study and chemical ingredients of the materials according to manufacturers’ information.

Brand Names (Code)	Manufacturer	Components	Primer/Adhesive	Processing Method
Silagum Comfort (SLC)	DMG, Hamburg, Germany	Vinyl polysiloxane, Hydrogen polysiloxane, aerosil, additives	Ethyl acetate, modified polyacrylate, additives Recommended for Silagum AM Comfort. Apply and let solvent dry for 1 min.	Autopolymerizing
Elite Soft Relining (ESR)	Zhermack Rovigo Italy	Vinyl polysiloxane (addition silicone) and platinum catalysts	Solution of polyacrylate in dichloromethane (Apply and let solvent dry for 1 min)	Autopolymerizing
Ufi Gel SC	VOCO GmbH Cuxhafen Germany	Modified polydimethylsiloxanes + platinum catalyst (addition silicone)	Butanone and methacrylates (Apply and let solvent dry for 60”)	Autopolymerizing
Mucopren Soft	Kettenbach GmbH Eschenburg Germany	Silicone polymers (Vinyl polysiloxane, Hydrogenpolysiloxanes) and fillers with platinum-catalyst (addition silicone)	Polymethyl methacrylate Copolymer in ethyl acetate Apply and let solvent dry for 90”.	Autopolymerizing

**Table 2:** Evaluating Cohen’s d effect size.

Cohen’s d	Interpretation
0.1	very small
0.2	small
0.5	medium
0.8	large
1.2	very large
2.0	huge

**Table 3:** Average tensile strength values for individual samples of tested materials.

System	Surface Finish	Seasoning Time	Sample Size [pcs]	Mean Value [N]	Variance [N]
Elite Soft	raw	24 h	15	172.6	49.32
	raw	6 weeks	13	145.9	73.50
	100 μm	24 h	11	125.0	60.38
	100 μm	6 weeks	8	168.2	28.20
	350 μm	24 h	7	162.6	29.08
	350 μm	6 weeks	7	140.0	34.34
Mucopren Soft	raw	24 h	14	63.2	14.45
	raw	6 weeks	11	56.3	8.046
	100 μm	24 h	8	47.1	9.69
	100 μm	6 weeks	15	54.1	12.43
	350 μm	24 h	8	56.8	8.8
	350 μm	6 weeks	13	45.9	8.09
Silagum Comfort	raw	24 h	10	153.5	38.62
	raw	6 weeks	9	163.6	54.23
	100 μm	24 h	7	159.9	74.57
	100 μm	6 weeks	8	220.6	45.77
	350 μm	24 h	11	204.7	32.35
	350 μm	6 weeks	10	141.4	59.81
Ufi Gel SC	raw	24 h	11	126.7	12.47
	raw	6 weeks	10	143.7	38.2
	100 μm	24 h	11	134.6	17.01
	100 μm	6 weeks	11	107.7	30.87
	350 μm	24 h	9	120.7	45.34
	350 μm	6 weeks	9	157.7	27.13

Where—[N]—force in Newtons; pcs = sample size.

**Table 4:** *p*-values for Kruskal–Wallis test used to check for same population distribution.

Lining System	*p*-Value
Elite Soft	0.013
Mucopren Soft	0.0002
Silagum Comfort	0.0002
Ufi Gel SC	0.0005

**Table 5:** *p*-values for one-tailed *t*-test used to check for the impact of seasoning time.

		Right-Tailed $H_{1}:\mu_{24h}>\mu_{6t}$	Left-Tailed $H_{1}:\mu_{24h}<\mu_{6t}$
	Surface Finish	*p*-Value	*p*-Value
Elite Soft	raw	0.127	0.873
	100 μm	0.961	0.039 *
	350 μm	0.105	0.895
Mucopren Soft	raw	1	0 *
	100 μm	1	0 *
	350 μm	1	0 *
Silagum Comfort	100 μm	0.334	0.666
	350 μm	0 *	1
Ufi Gel SC	100 μm	0.194	0.807
	350 μm	0.998	0.002 *

[*] statistically significant.

**Table 6:** The effect size for *t*-test verifying the influence of seasoning time (24 h vs. 6 weeks) on glueing joint durability.

	Surface Finish	Cohen’s d	Interpretation
Mucopren	raw	0.6	medium
	100 μm	−0.7	medium
	350 μm	1.4	very large
Silagum	350 μm	1.5	very large
Ufigel	350 μm	−1.1	large

**Table 7:** *p*-values from ANOVA and *t*-test used to check if an increased roughness increases the joint durability.

	Seasoning Time	*p*-Value	test
Elite Soft	24 h	0.067	ANOVA
	6 weeks	0.572	ANOVA
Mucopren Soft	24 h	0.019 *	ANOVA
	6 weeks	0.031 *	ANOVA
Silagum	24 h	0.047 *	ANOVA
	6 weeks	0.007 *	Student’s *t*-test
Ufi gel	24 h	0.526	ANOVA
	6 weeks	0.001 *	Student’s *t*-test

[*] statistically significant.

**Table 8:** *p*-values from one-tailed *t*-tests used to check the impact of surface roughness with regard to seasoning time.

		Right-Tailed $H_{1}:\mu_{1}>\mu_{2}$	Left-Tailed $H_{1}:\mu_{1}<\mu_{2}$
	(1)–(2)	*p*-Value	*p*-Value
Mucopren			
24 h	raw–100 μm	0.006 *	0.994
	raw–μm	0.133	0.867
	100 μm–350 μm	0.972	0.028
6 weeks	raw–100 μm	0.304	0.696
	raw–350 μm	0.002 *	0.998
	100 μm–350 μm	0.026 *	0.974
Silagum			
24 h	raw–100 μm	0.591	0.409
	raw–350 μm	0.998	0.002 *
	100 μm–350 μm	0.952	0.048 *
6 weeks	100 μm–350 μm	0.004 *	0.996
Ufi Gel			
6 weeks	100 μm–350 μm	0.999	0.001 *

[*] statistically significant.

**Table 9:** The *t*-test effect size for verifying the influence of surface finish on glued joint durability.

	(1)–(2)	Cohen’s d	Interpretation
Mucopren			
24 h	raw–100 μm	1.2	very large
	100 μm–350 μm	−1.1	large
6 weeks	raw–350 μm	1.3	very large
	100 μm–350 μm	0.8	large
Silagum			
24 h	raw–350 μm	−1.4	very large
	100 μm–350 μm	−0.9	large
6 weeks	raw–100 μm	1.5	very large
Ufi Gel			
6 weeks	100 μm–350 μm	−1.7	very large

## Data Availability

The raw data supporting the conclusions of this article will be made available by the authors on request.
